# The Influence of Perceptual Training on Working Memory in Older Adults

**DOI:** 10.1371/journal.pone.0011537

**Published:** 2010-07-14

**Authors:** Anne S. Berry, Theodore P. Zanto, Wesley C. Clapp, Joseph L. Hardy, Peter B. Delahunt, Henry W. Mahncke, Adam Gazzaley

**Affiliations:** 1 Departments of Neurology and Physiology, W.M. Keck Foundation Center for Integrative Neuroscience, University of California San Francisco, San Francisco, California, United States of America; 2 Posit Science Corporation, San Francisco, California, United States of America; University of Sydney, Australia

## Abstract

Normal aging is associated with a degradation of perceptual abilities and a decline in higher-level cognitive functions, notably working memory. To remediate age-related deficits, cognitive training programs are increasingly being developed. However, it is not yet definitively established if, and by what mechanisms, training ameliorates effects of cognitive aging. Furthermore, a major factor impeding the success of training programs is a frequent failure of training to transfer benefits to untrained abilities. Here, we offer the first evidence of direct transfer-of-benefits from perceptual discrimination training to working memory performance in older adults. Moreover, using electroencephalography to evaluate participants before and after training, we reveal neural evidence of functional plasticity in older adult brains, such that training-induced modifications in early visual processing during stimulus encoding predict working memory accuracy improvements. These findings demonstrate the strength of the perceptual discrimination training approach by offering clear psychophysical evidence of transfer-of-benefit and a neural mechanism underlying cognitive improvement.

## Introduction

Computerized training programs are increasingly being developed to improve perception, attention and memory abilities in older adults [1], [2]. One cognitive training approach has been to induce perceptual learning in trainees via repetitive exposure to sensory stimuli in the setting of adaptively challenging stimulus discrimination tasks [3]. Perceptual learning in the visual domain has been documented to occur in young adults [4], [5], [6], [7], although it has been shown to be specific for the relevant stimulus features in the task being practiced, such that there is limited improvement in discrimination of features that differ by orientation [8], [9], [10], spatial frequency [10], direction of motion [4], [11] or visual field location [9], [10], [12], [13]. Although perceptual learning has also been documented to occur in older adults [14], [15], the ability, or inability, of discrimination training to transfer benefits to different perceptual features has not yet been evaluated. It is reasonable that such transfer may occur in a population that has baseline perceptual impairment, such as older adults [3].

Working memory (WM) abilities are diminished in older adults relative to performance of younger adults [16]. Aspects of age-related decline in higher cognitive functions such as WM may be related to deficits in perception [17], [18], although impairment has been shown to exist independent of perceptual differences [19]. We hypothesize that training programs that are successful in improving perceptual abilities in older adults will also have direct consequences on higher cognitive functions, such as WM, via the generation of higher fidelity internal representations of to-be-remembered stimuli [2]. It is critical to investigate the factors that facilitate generalization of training-induced benefits, so as to improve the efficacy of programs targeting cognitive decline in older populations.

**Figure 1:**
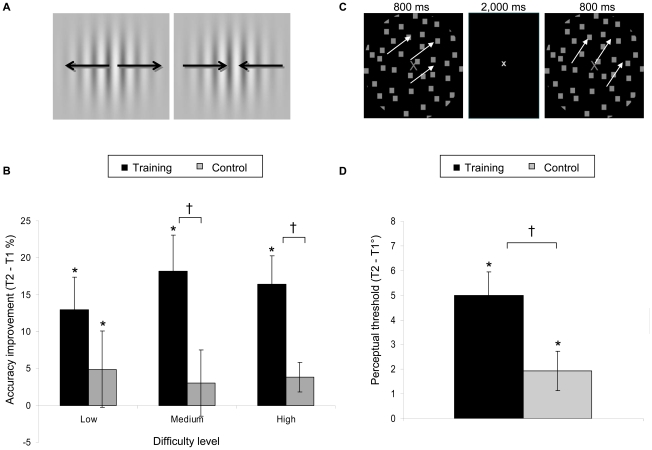
Perceptual discrimination. 1a. Perceptual discrimination training paradigm. Gabor pattern filters required a discrimination judgment of whether the stimuli expanded or contracted. Training was adaptive, such that changes in the ISI and stimuli duration scaled with performance. 1b. Training effects on trained task. Trained older adults showed significant improvement over untrained controls at medium and high difficulty tasks (100 ms and 50 ms stimuli and ISI duration, respectively). 1c. Untrained perceptual discrimination paradigm. Discrimination thresholds for direction of 100% coherent motion was tested using moving dot kinetograms. Participants made a judgment as to whether two presented directions of motion matched one another. 1d. Training effects on untrained task. Perceptual discrimination thresholds of trained older adults improved significantly more than untrained controls. * Asterisks indicate significant differences within group from T1 to T2 (paired t-test). Crosses indicates significant group by time interactions (repeated measures ANOVA).

To examine the behavioral and neural effects of perceptual discrimination training on a distinct perceptual task, as well as WM performance, in healthy older adults, we evaluated two groups of 15 participants (ages 60–89 years) before (T1) and after (T2) either ten hours of visual discrimination training over a three to five week period (training group) or no training (control group). Stimuli used in the training program were Gabor patterns (sine-waves windowed by a 2D Gaussian), which expanded or contracted two successive times per trial (Fig. 1a). Participants pressed one of two buttons for each movement to indicate whether they perceived the stimuli expanding or contracting. Training was adaptive such that the speed of expansion/contraction and the duration of the inter-stimulus interval (ISI) scaled with improvements in response accuracy, so as to continuously challenge the trainees [20]. Differing colors and orientations of training stimuli varied across trials to facilitate generalization. Changes in perception, attention and WM performance on untrained tests were assessed for both groups to evaluate transfer-of-benefit, and simultaneous electroencephalography (EEG) recordings were utilized to assess neural mechanisms of training-induced performance changes.

## Results

### Neuropsychological Assessment

Baseline neuropsychological measures confirmed that participants showed normal cognitive performance. MMSE scores ranged from 27–30 (mean = 29.3, SE = 0.16) with no significant difference between training and control groups (t(28)  = 0.62, p = 0.87). Performance on NeuroTrax measures of global cognition, memory, executive function, attention, and information processing speed were within 2 SD of age and education matched normative values for every participant for every measure with no significant differences across groups (all t(28) >0.69, p>0.59).

### Behavioral performance on trained perceptual task

Consistent with findings from perceptual learning studies in young adults [21], the performance of older adults in the training group significantly improved on the discrimination task, relative to the untrained control group. Training and control groups showed comparable performance for the trained task at T1 (all main effects of group: F<2.23, p>0.15). Trained participants showed significant improvement at T2 versus T1 in both speed threshold on an adaptive test version of the trained task, and detection accuracy on a fixed-speed test version of the trained task (all time by group interactions: F>5.18, p<0.05). Of note, accuracy improvement on the fixed-speed test was significant only for medium and high difficulty levels of the task (i.e., 100 ms and 50 ms stimuli and ISI duration, respectively) and not the low difficulty level (200 ms stimuli and ISI duration)(Fig. 1b). This result supports previous evidence of perceptual learning in older adults with challenging discrimination practice [22].

### Behavioral performance on untrained perceptual task

To test whether discrimination training on the trained task generalizes to improvements in untrained perceptual abilities in older adults, both groups were tested on a perceptual discrimination task at T1 and T2 for the direction of motion of random dot kinetograms (Fig. 1c). A single direction of 100% coherent motion was presented followed by a second direction (fixed ISI  = 2 sec) and participants indicated whether they perceived the two directions as the same or different by pressing one of two buttons. Each participant's discrimination threshold was determined using a stair-step procedure (2° step). This discrimination threshold was later used to determine the perceptual difficulty of the working memory task (described below) for each participant. T1 baseline measures revealed that perceptual discrimination abilities of older adults were significantly reduced compared to younger adults (t(48)  = 3.83, p<0.001), who performed an identical task in a recent study [23]. This is consistent with perceptual deficits that are known to occur with normal aging [24]. T1 perceptual thresholds were comparable for training and control groups with no significant main effect of group (F(1,28)  = 0.06, p = 0.80) (training mean = 28.33, SE = 1.78; control mean = 27.27, SE = 1.95). However, training significantly improved perceptual discrimination on this untrained task relative to age-matched controls (time by group interaction; (F(1,28)  = 8.40, p<0.01, effect size d = 0.91) (Fig. 1d). Furthermore, individual improvement on the trained task correlated with discrimination improvements on the untrained task (r = 0.46, p<0.05, Figure S1). Together, these results provide evidence of transfer of training benefits to an untrained perceptual task in older adults.

### Behavioral performance on the untrained working memory task

**Figure 2:**
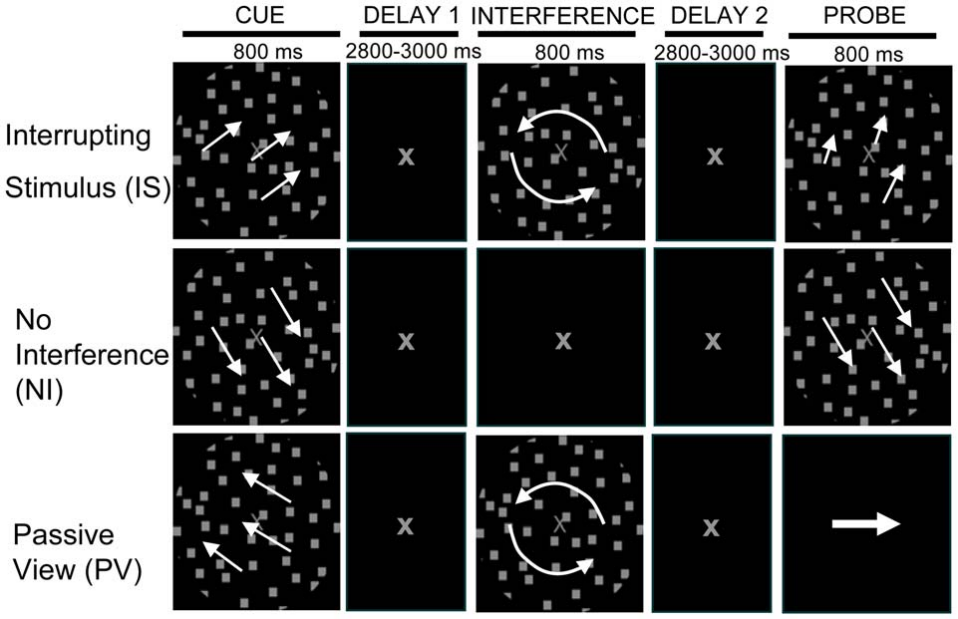
Working memory paradigm. Delayed-recognition paradigm. Working memory for the direction of 100% coherent motion was tested using moving dot kinetograms in two tasks. Participants encoded a direction of motion (cue) and after a delay period determined if the probe direction matched the cue direction. In the Interrupting stimuli task (IS), a circular swirl of motion was presented in the middle of delay period. A button press was required if the swirl was fast. A third task was perceptually equivalent to the WM tasks, but participants were instructed to passively view stimuli and press a right or left button at probe depending on the direction of an arrow.

To test whether perceptual discrimination training results in improvement on higher-level cognitive operations, working memory (WM) for the direction of motion of moving random dot kinetograms was evaluated in two delayed-recognition tasks: No Interference (NI) and Interrupting Stimulus (IS) (Fig. 2). For the NI task, a motion stimulus to be remembered was presented, followed by a delay period, and then a probe motion stimulus, which was either identical to the original motion direction or differed by a vector angle equivalent to the participant's pre-determined perceptual discrimination threshold. Participants pressed one of two buttons to indicate whether the directions were the same or different. In the T2 assessment, the vector angle of the probe was determined using both the participant's original threshold at T1 and the post-training threshold at T2 (new threshold) in separate experimental blocks. The IS task was identical except that a circular motion stimulus was presented in the middle of the delay period, and required a simple perceptual discrimination (multi-tasking manipulation). In a Passive View task (PV), participants were instructed to merely view the stimuli and make a button press response at probe to indicate the direction of an arrow (left or right). At baseline testing (T1), WM accuracy was equivalent between the two older groups (no main effect of group: F(1,28) <0.001, p = 0.95) and was significantly impaired by the presence of the secondary task (IS) in both groups (main effect of task: F(1,28)  = 5.63, p<0.05). Of note, WM performance was also significantly impaired relative to younger adults who performed identical versions of the delayed-recognition tasks in a single EEG session without training for another study [23]. This age-related deficit existed despite perceptual difficulty being equilibrated across age groups using the thresholding procedure (main effect of age: F(1,48)  = 8.46, p = 0.005).

**Figure 3:**
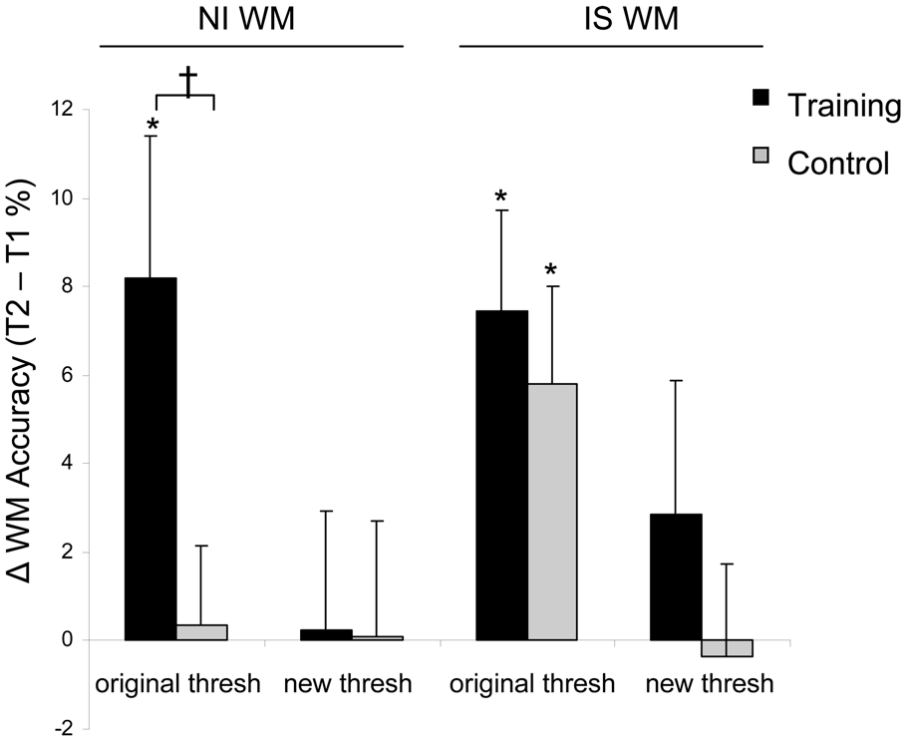
Working memory performance. At T2, WM was tested on the NI and IS tasks at the participant's original perceptual threshold (obtained at T1) and new threshold (obtained at T2). Change in WM accuracy was calculated as T2 – T1. Training led to WM improvement in the NI task compared to controls when tested at their original threshold (training effect). Neither group showed changes in WM performance when tested at their new perceptual threshold. Both groups improved in the IS task at the original threshold (practice effect), but not at the new threshold. * Asterisks indicate significant differences within group from T1 to T2 (paired t-test). Crosses indicates significant group by time interactions (repeated measures ANOVA).

Evaluation of WM performance on the NI task at the original discrimination threshold revealed a training effect, such that only the training group exhibited significantly improved WM accuracy at T2 vs. T1 (time by group interaction; (F(1,28)  = 4.982, p<0.05, effect size d = 0.81) (Fig. 3). Thus, we show for the first time in any age group that perceptual discrimination training can result in improved WM performance on an untrained task. Remarkably, after 10 sessions of training, the older adult post-training performance reached the accuracy levels of younger adults without training (t(33)  = 0.26, p = 0.79), demonstrating that aspects of age-related impairment in cognitive performance can be improved via perceptual training.

One potential mechanism for this effect is that improved WM performance is a direct consequence of an enhanced ability of participants to generate high-fidelity internal representations of the encoded stimuli during presentation [2]. A modest correlation between improvements in perceptual discrimination threshold and WM accuracy supports this conclusion (r = 0.43, p = 0.05, 1-tailed, Figure S2). To evaluate this further, we tested training-related WM improvement independent of perceptual gains by assessing changes in WM performance using each participant's new perceptual threshold at T2 (13/15 participants in the training group showed decreased thresholds at T2, i.e., improved discrimination). This analysis revealed that WM performance in the training and control groups did not significantly improve when tested at the new threshold (no main effect of time: F(1,28)  = 0.02, p = 0.89; no group by time interaction: F(1,28)  = 0.02; p = 0.90)(Fig. 3). Thus, normalizing perceptual demands to take into account improved perceptual abilities after training eliminates training-related facilitation of WM performance, supporting the hypothesis that perceptual enhancement in the training group drives the WM performance improvement.

WM performance was evaluated for the IS task at the participant's original perceptual threshold. Comparison of T1 and T2 accuracy revealed a practice effect, such that both training and control groups significantly improved their WM accuracy (main effect of time: F(1,28)  = 17.84, p<0.001), whereas no training effect was observed, i.e., no significant difference between the groups (no time by group interaction: F(1,28)  = 0.35, p = 0.56) (Fig. 3). This is consistent with previous findings in younger and older adults of practice-related improvements in WM performance in the setting of external interference, even over the course of a one-hour session [23], [25]. However, it is important to note that perceptual discrimination training did not ameliorate the negative impact of interference on WM performance beyond that attained by limited practice on the task. There was no practice or training effect for the IS task at the new threshold (no main effect of time: F(1,28)  = 0.57, p = 0.46; no time by group interaction: F(1,28)  = 0.80, p = 0.38).

### Electroencephalography

**Figure 4:**
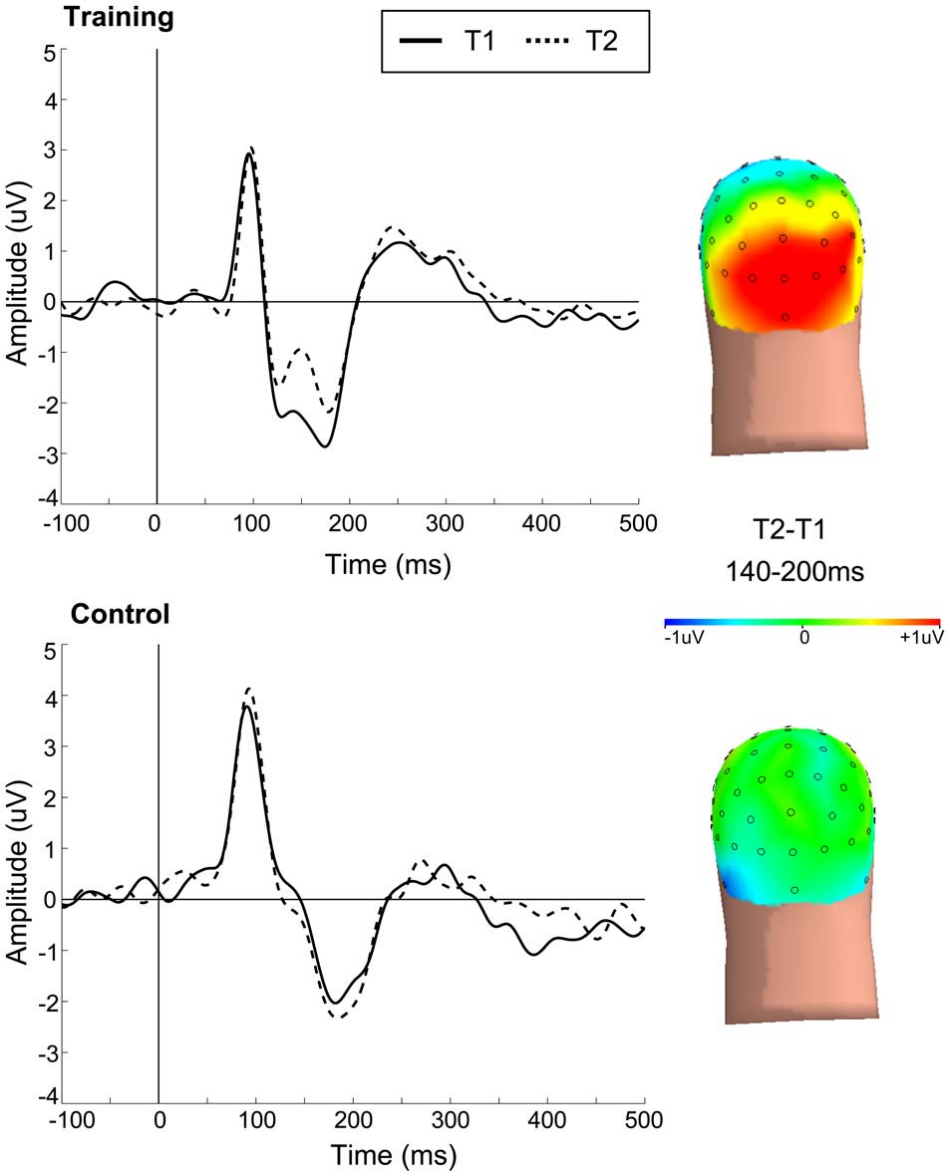
EEG Recordings. Event-Related Potentials during stimulus encoding. Posterior occipital N1 amplitude (120–220 ms) significantly decreased at T2 for the training, but not control group. Statistics are based on electrode of interest (EOI) clusters selected for each participant. Scalp topographies of T2-T1 at the latency of mean N1 peak +/− 1sd illustrate the location of the training related functional plasticity.

**Figure 5:**
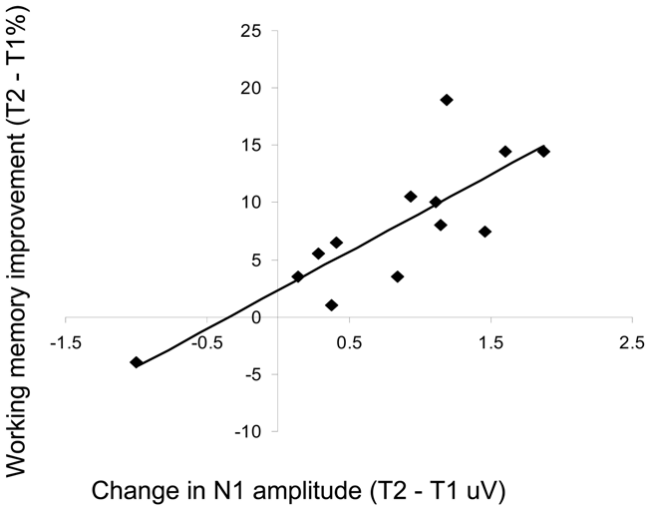
Neural-behavioral correlation. Across participants, decreased N1 amplitude during encoding correlated with WM performance improvements in the NI task at the original threshold in the training group (r = 0.82, p<0.001).

To explore the underlying neural mechanisms of successful transfer of perceptual training to WM performance gains, we recorded event-related potentials (ERP) during the WM experiments at both T1 and T2 (each group, N = 13). Analysis focused on the N1 posterior visual ERP time-locked to encoding, interference, and probe stimuli. The N1, a negative deflection occurring between 140 ms and 220 ms, is a marker of early visual processing of motion direction [26], with its anatomical source localized to a network of visual cortical areas, including the middle temporal area MT+/V5 [27], [28]. N1 amplitudes at T1 were not significantly different for training and control groups (no main effect of group: F(1,24)  = 1.37, p = 0.25). Evaluation of the ERP for the encoding stimuli in the NI task at the original threshold (i.e., the task that exhibited a WM training effect), revealed a significant decrease in N1 amplitude at T2 for the training group, but not the control group (time by group interaction: F(1,24)  = 15.42, p<0.001) (Fig. 4). Moreover, there was a strong correlation between improved WM accuracy and changes in N1 amplitude with training (r = 0.82, p<0.001) (Fig. 5), suggesting that diminished N1 amplitude generated by the encoded stimuli is a predictor of training-related WM gains. This is consistent with our hypothesis that training-induced changes in the visual processing of encoded stimuli yield improved WM performance.

When interpreting the functional significance of the reduced N1 amplitude after training, it is important to consider that the N1 amplitude is modulated by attention [29], and so it is possible that reduced demands of an “easier” post-training task may have resulted in less attentional effort during encoding and thus a decreased neuronal response [30]. To examine this possibility, we evaluated the N1 amplitude for the other WM tasks, notably the tasks performed at the new threshold, in which there were no observed WM improvements (i.e., equivalently challenging at T1 and T2). We found decreased N1 amplitudes at T2 versus T1 only in the training group for encoded stimuli in the NI task at the new threshold, and the IS task at the original and new threshold, (all time by group interactions: (F(1,24) >4.48, p<0.05)). This three-fold replication of the N1 training effect illustrates that it is not simply the reflection of a change in attention mediated by task demands, but reveals a training-induced modification in neural response to behaviorally relevant motion stimuli. Interestingly, passively viewed stimuli (PV) did not show N1 amplitude changes after training, suggesting that attention is necessary for training-related effects on visual processing.

No significant changes in the N1 amplitude time-locked to the probes were identified in the training or control groups for NI, IS and PV tasks (all group by time interactions F<1.90, p>0.18). A comparable analysis was performed for the P1 component, a positive deflection occurring between 60–140 ms, with no observed differences across groups or time during encoding or probe periods for NI, IS, and PV tasks. (all group by time interactions: F<1.50, all p>0.21).

Although N1 amplitude changes during encoding predicted improved WM performance in the NI task, this was not true for the IS task. For this task, successful WM performance was mediated by effective processing of interrupting stimuli, rather than the representation of the encoded stimuli. A previous study in younger adults showed that attentional modulation of interrupting stimuli in this same task, as revealed by N1 amplitude indices, predicted WM performance and decreases in enhancement of these stimuli with practice over a single session predicted improvements in WM [31]. Consistent with these findings, older adults showed a significant decrease in enhancement of interruptors during T2 (p<0.05). While this evidence of practice-related changes in interference processing in older adults after limited exposure to a task is encouraging, it is important to note that improvements in WM performance by practice was limited, as revealed by interference still disrupting WM performance at T2 relative to non-interference levels (NI task) after training (t(14)  = 2.65, p<0.05).

## Discussion

This study offers critical evidence of the benefit that perceptual training has on visual perception and WM performance in older adults. We present neural data of training-related plasticity in older adults and identify a neural marker of perceptual training transfer that correlates with WM performance enhancement. We propose that the N1 amplitude decrease is a neural marker of the perceptual gains induced by training, which then engenders WM improvement.

These findings are consistent with animal models of perceptual learning showing that tuning curves become narrower for the trained population of visual cortical neurons [32], and that as neurons narrow their tuning curves, smaller responsive neural populations are reflected by decreased EEG [33], [34] and fMRI [35] signals in humans. Further, the observed plasticity in this study occurs in neural populations higher than V1, consistent with the notion that stimulus specificity during learning is targeted to the level of visual processing demanded by the task [36]. The absence of a training-related neural change at the P1, which is temporally earlier than the N1 and reflects activity in earlier visual cortical areas, supports this hypothesis.

This study validates the use of perceptual discrimination training in older adults to improve WM performance, and highlights the need for the continued development and rigorous evaluation of training programs targeting deficient processes. Perceptual discrimination training did not improve WM performance beyond control practice levels when the delayed-recognition task was interrupted by another task. This suggests that training specifically directed at interference processing may be necessary to mitigate the negative impact multi-tasking has on WM performance. Additionally, when training-related perceptual improvements were normalized by discrimination thresholding, the training group did not exhibit improved WM accuracy and continued to show impaired WM performance relative to younger adults (main effect age (1,33)  = 3.07, p = 0.09) [31]. These results reveal that WM deficits with aging cannot be corrected solely by remediating age-related perceptual impairment, but perhaps by also targeting interventions at other processes supporting WM, such as memory maintenance.

## Materials and Methods

### Ethical Statement

Participants were paid for their participation and gave informed written consent. The Committee on Human Research at the University of California, San Francisco, approved the EEG portion of the study. The cognitive training portion of the study received separate approval by an independent IRB review board (Independent Review Consulting Incorporated, Corte Madera, CA).

### Participants

32 healthy older adults (mean age 71.93, SE 1.33; 18 females) were recruited and randomly assigned to control and training groups after consent. Two participants were enrolled, but did not complete the study because of unwillingness to participate in the final EEG session. Statistics reported reflect 30 participants who completed 10 sessions of training and both EEG sessions. Participants had 13–21 years of education (mean = 17.24 years, SD = 2.32), with no significant difference across groups (t(28)  = 0.979, p = 0.34). All participants had normal or corrected-to-normal vision, did not have a history of stroke, traumatic brain injury, psychiatric illness, or previous experience with visual cognitive training. Participants did not take psychotropic medication. Participants were characterized as cognitively normal using standard neuropsychological assessment conducted prior to study initiation.

All participants were from the San Francisco Bay Area and recruited using a database of research volunteers at Posit Science, which was previously compiled using local advertisements and mailings. Contraindications were screened for during a standardized phone interview. Participants were randomized to training or control groups after signing consent forms for participation in the study at Posit Science offices. Experimenters from the University of California, San Francisco who conducted the behavioral and EEG analysis were blinded until after data for the final group analysis was completed.

Performance of the older adults was compared to a group of 20 younger participants (mean age 24.2, SE 0.49; 9 females) who engaged in the untrained perceptual and working memory tasks without training during a single EEG session for another study [23].

### Neuropsychological Assessments

Baseline neuropsychological measures were collected for each participant to confirm that cognitive performance was within two standard deviations of the normative values for their age and education. Mini-mental state exam (MMSE) (all MMSE scores were greater than 27) and NeuroTrax (Mindstreams) measures of global cognition, memory, executive function, attention, and information processing speed were completed by all participants. NeuroTrax has been validated for use as an assessment for the detection of possible mild cognitive impairment [37], [38].

### Training

Participants in the training group completed 10 hours of visual cognitive training using the Sweep Seeker program (InSight, Posit Science). Sweep Seeker training is a stand-alone module in the Posit Science InSight software package. The perceptual training exercise was embedded in a block type game to encourage attention, provide feedback and rewards, and improve compliance for the 10 hours of training. Additionally, the software was designed to be easy to use, so that previous experience with computers would not limit the population that may benefit from such a cognitive training approach. Training took place in 40-minute sessions, 3–5 sessions/week for 3–5 weeks. Training occurred in either in research offices (n = 6) or at home (n = 9) where computer equipment was provided to participants. Participants did not have the option of doing some training in home and some at the research offices. There were no location-dependent differences in trained task performance measured by repeated measures ANOVA with factors location (home vs. office) and time (pre-training performance vs post-training performance) as indicated by no location X time interaction (F(1,13)  = 0.89, p = 0.36). The data from each training session was automatically uploaded to remote servers, providing a complete record of program usage (e.g., days trained, total training time) and progress (e.g., stimulus challenge level). Participants were phoned regularly to encourage compliance.

Each trial consisted of two sweeping Gabor pattern stimuli (sine-wave patterns windowed by a 2D Gaussian) (**Figure 1a**). The patterns either expanded or contracted across a range of spatial frequencies (0.50 to 5.00 cycles per degree) and subtended 8 degrees of visual angle. The stimulus presentation time and ISI were adjusted together using an adaptive staircase algorithm (ZEST) [20]. Differing colors and orientations of sweeps varied training conditions. Vertical, horizontal, and diagonal orientations were utilized in distinct blocks. Steps were taken to assure that training conditions at home and in the office were standardized by calibrating stimuli to accurately specify chromaticities and relative luminances on home computers. Participants indicated the sequence of stimulus presentation by clicking on icons presented after each trial. All training was performed at the 85% correct level of the psychometric function estimated by the ZEST algorithm. Thresholds were calculated by taking the log mean of two randomly interleaved staircases.

### Untrained Perceptual and Working Memory Task Stimuli

The stimuli consisted of a circular aperture containing 290 dots (0.08°×0.08° each) that subtended 8° of visual angle at a 75 cm viewing distance and were centered at the fovea as previously described [23].

### WM Experiment with EEG

#### Stimuli

The stimuli consisted of a circular aperture containing 290 dots (0.08°×0.08° each) that subtended 8° of visual angle at a 75 cm viewing distance and were centered at the fovea. This field of 290 spatially random gray scale dots moved with 100% coherence at an oblique angle at 10° per second. Stimuli were presented with a gray fixation cross in the center of the circular aperture with a black background of luminance level 0.32 cd/m^2^. All four sectors of the aperture were used (i.e. northeast, northwest, southeast, southwest) except the cardinal directions (up, down, left, right) [39]. The experimental stimuli consisted of 12 different directions of motion (3 per sector). Stimuli were presented through E-Prime software (Psychology Software Tools, Inc.) run on a Dell Optiplex GX620 and a ViewSonic G220fb CRT monitor.

#### Thresholding

Participants completed a motion thresholding test prior to the onset of the main experiment in order to minimize the effects of individual differences in discriminability. A staircase procedure (2° increments) required participants to determine whether two motion stimuli were moving in the same direction. The two 100% coherent motion stimuli were presented for 800 ms each and separated by 2000 ms (**Figure 1c**). An angle of discrimination (the difference between two directions of motion) was selected for each participant as the largest angle at which discrimination performance was less than 100%.

#### Experimental Procedure

In a paradigm previously described [23], participants were presented with three different tasks randomized across six blocks, with two blocks per task **(** **Figure 2** **)**. There were two WM tasks: Interrupting Stimulus (IS), and No Interference (NI). A third task instructed participants to passively view the stimuli (PV). At T1, participants completed another WM task, Distracting Stimulus (DS), which was not completed at T2 because of experimental time constraints.Results are not discussed here.

#### Data Acquisition

Participants sat in an armchair in a dark, sound-attenuated room for neural recordings. Data were recorded during blocks (two blocks per task condition) lasting approximately 8 minutes each and a total of 80 trials per task. Electrophysiological signals were recorded with an ActiveTwo BioSemi 64-channel Ag-AgCl active electrode EEG acquisition system in conjunction with ActiView software (BioSemi). Signals were amplified and digitized at 1024 Hz with a 24-bit resolution. All electrode offsets were between +/−20 mV. Anti-aliasing filters were used during data acquisition, and the data were referenced to the average offline. Precise markers of stimulus presentation were acquired using a photodiode.

#### Data Analysis

EEG preprocessing: Eye movement artifacts were removed using Brain Vision Analyzer (Brain Products GmbH) through an independent component analysis (ICA). Only ICA components consistent with topographies for eye blinks and horizontal eye movement were removed. Additionally, individual trials containing artifacts with a voltage threshold of ±50 µV were removed. Data were band-pass filtered between 1–30 Hz.

#### EEG analysis

A 200 ms pre-stimulus baseline was subtracted from each trial prior to calculating the evoked-response potential (ERP). ERP peaks were obtained from posterior scalp sites over pre-selected latency ranges (P1 range: 60 ms–140 ms; N1 range: 120 ms–220 ms). Trials were averaged into task and block-specific grand average ERPs for each participant. ERP statistical analysis used an electrode of interest (EOI) method [23], [26], [40], [41], [42], [43], [44], [45], [46]. A unique electrode was selected for each participant and averaged with 3–4 surrounding electrodes for use in group-level statistics. EOIs were defined for each participant as the posterior electrode whose grand average of all tasks averaged together (IS, NI, and PV) had the largest ERP peak amplitude. This method is designed to identify the electrode most sensitive to the neural responses associated with the task stimuli. EOIs for interfering stimuli were selected independently from cue and probe EOIs. Separate EOIs were selected for P1 and N1 peaks. EOIs were selected from the following posterior electrodes for P1: P9, P7, P5, P6, P8, P10, PO7, PO3, POz, PO4, PO8, O1, Oz, O2, Iz. Posterior midline electrodes were not included for N1 EOI selection. EOI selection was made independently for T1 and T2 recording sessions. If a participant's EOI across sessions was not the same or neighboring electrode, the EOI was selected from the grand average of T1 and T2 sessions. EEG data from 4 participants (2 training, 2 control) were not included in analysis due to equipment changes across test sessions.

ERP statistical analysis was performed using two-way ANOVA with factors of group (training vs. control) and time (T1 vs. T2) for each task condition. The Greenhouse-Geisser correction was applied when sphericity was violated. Significant main effects and interactions were evaluated with post-hoc t-tests with false discovery rate (FDR) correction for multiple comparisons [47]. Cohen's d effect size and two-tailed Pearson's correlations were also calculated.

### Behavioral analysis

RTs from the passive baseline task were subtracted from RTs from the WM tasks to account for individual differences in motor speed. These motoric speed-corrected RTs are referred to as the “RT index.”

## Supporting Information

Figure S1 Correlation of performance gains on trained and untrained visual perception discrimination tasks. Perceptual improvement on the trained Sweeps Seeker task correlates with perceptual improvement on the untrained motion direction task (training group: r = 0.46, p<0.05).
(0.42 MB TIF)

Figure S2 Correlation of performance gains in perceptual threshold task and WM task (No interference). Perceptual threshold improvement on the correlates with NI working memory improvement at original threshold (T2 NI original - T1 NI) (training group: r = 0.43, p = 0.05, 1-tailed).
(0.42 MB TIF)
