# Supplementary figures and images for: The Influence of Perceptual Training on Working Memory in Older Adults
Source: PLoS One. 2010 Jul 14;5(7):e11537. doi: 10.1371/journal.pone.0011537 (PMC2904363; doi:10.1371/journal.pone.0011537)

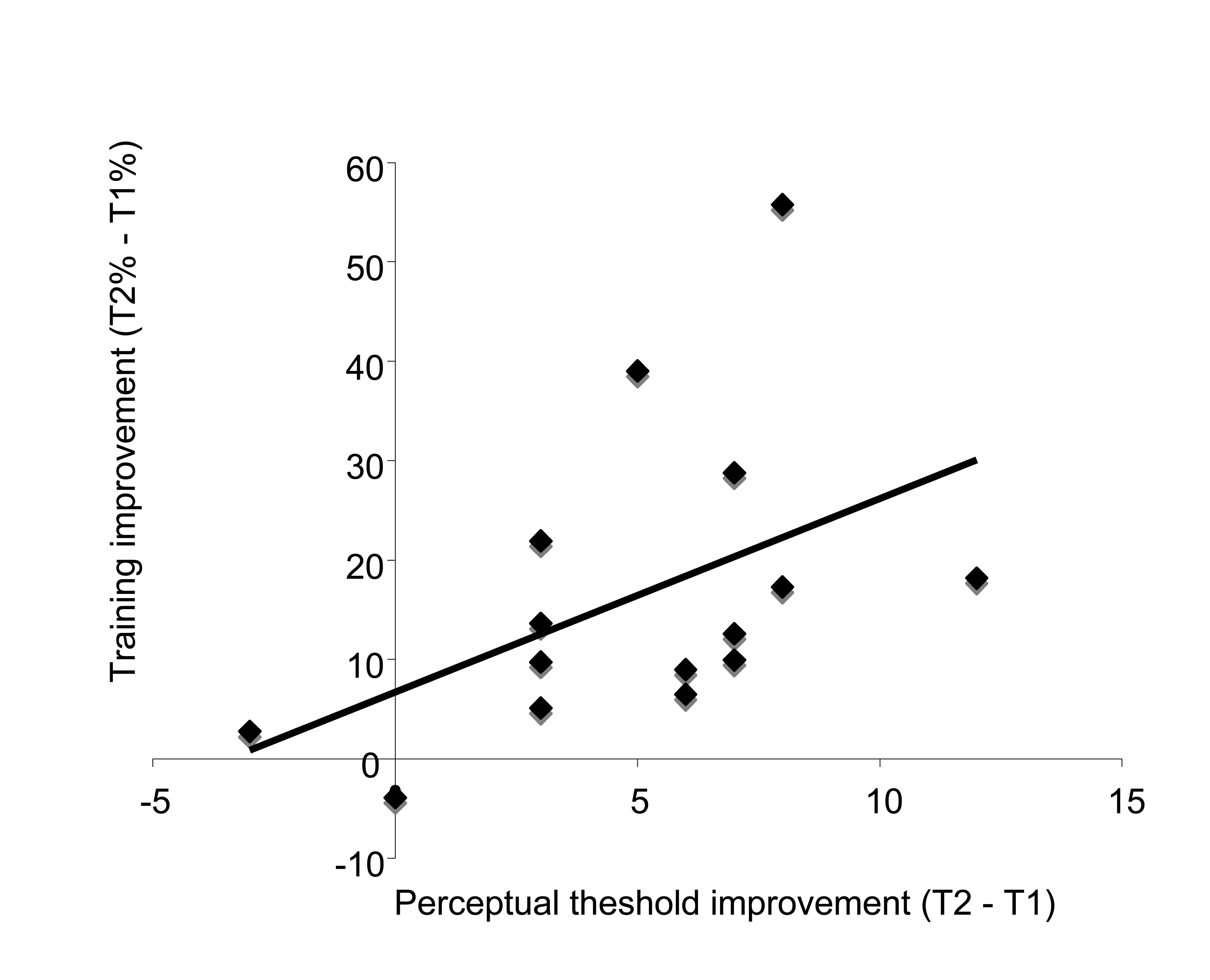

Supplement: Figure S1 — Correlation of performance gains on trained and untrained visual perception discrimination tasks. Perceptual improvement on the trained Sweeps Seeker task correlates with perceptual improvement on the untrained motion direction task (training group: r = 0.46, p<0.05). (0.42 MB TIF) [file pone.0011537.s001.tif]

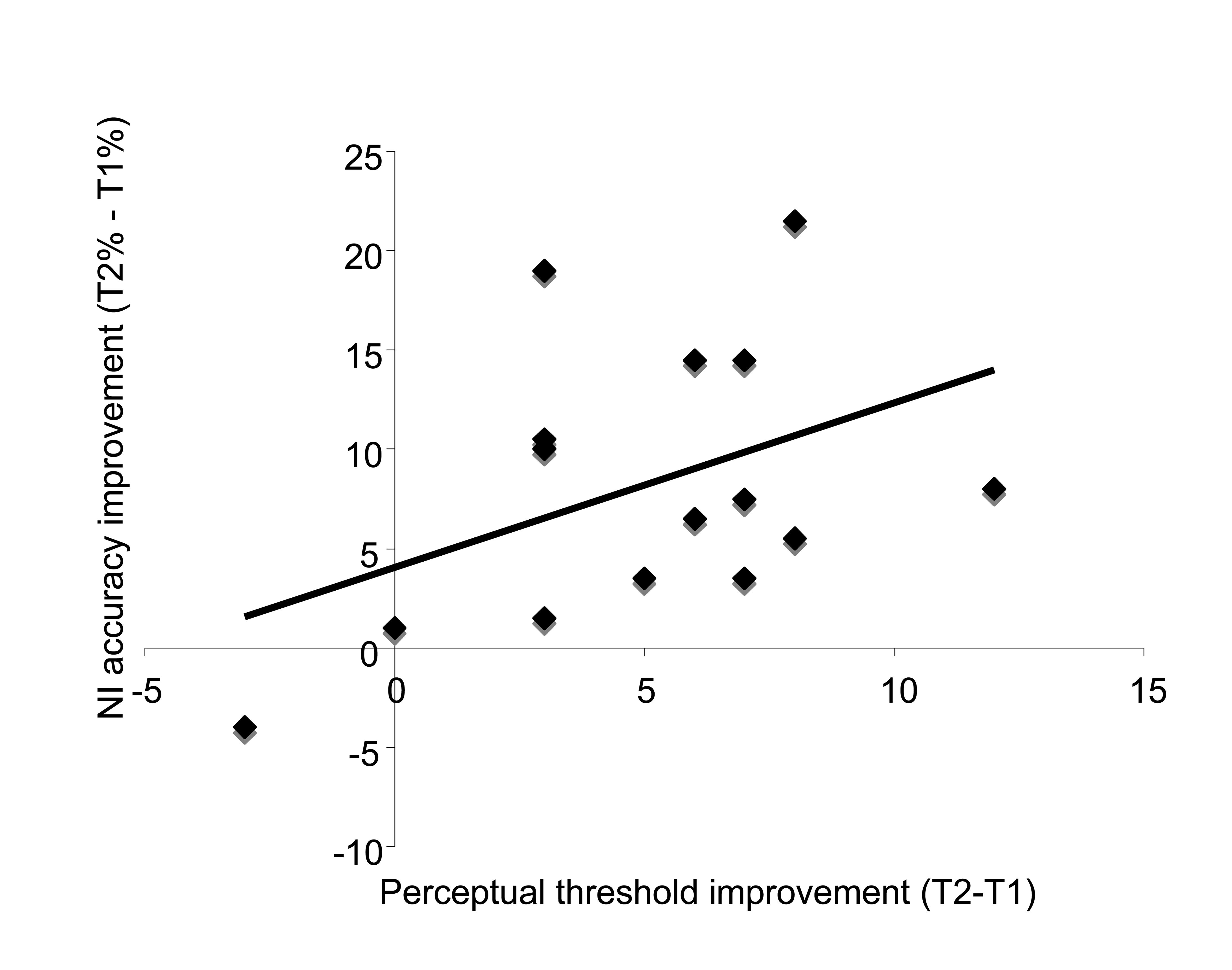

Supplement: Figure S2 — Correlation of performance gains in perceptual threshold task and WM task (No interference). Perceptual threshold improvement on the correlates with NI working memory improvement at original threshold (T2 NI original - T1 NI) (training group: r = 0.43, p = 0.05, 1-tailed). (0.42 MB TIF) [file pone.0011537.s002.tif]
